# Editorial: Cross-Disciplinary Perspectives on the Relationship Between Humor and Health: Theoretical Foundations, Empirical Evidence and Implications

**DOI:** 10.3389/fpubh.2021.774353

**Published:** 2021-10-25

**Authors:** Florian Fischer, Corinna Peifer, Tabea Scheel

**Affiliations:** ^1^Institute of Public Health, Charité – Universitätsmedizin Berlin, Berlin, Germany; ^2^Institute of Gerontological Health Services and Nursing Research, Ravensburg-Weingarten University of Applied Sciences, Weingarten, Germany; ^3^Research Group Work and Health, Department of Psychology, University of Lübeck, Lübeck, Germany; ^4^Department of Work and Organizational Psychology, International Institute of Management and Economic Education, Europa-Universität Flensburg, Flensburg, Germany

**Keywords:** health communication, mass media, prevention, emotion, positive psychology

## Introduction

Humor is a ubiquitous phenomenon in our daily lives. People might be exposed to it at a diversity of times and places from social interactions to medial representations (1). Humor is also an integral part of aspects which are directly or indirectly related to health and well-being (2, 3).

Based on previous research, we can understand humor as a personality tendency referring to a sensation (amusement), to a behavior (laughter or smiling), to its use as a way of coping (humor as a coping mechanism), to an ability (production and creation of stimuli) and finally, to an aesthetic sense (sense of humor, appreciation of humor) (4). Having a good sense of humor is thought to be a healthy and desirable personality trait as it refers to the readiness to respond positively to serious, uncomfortable and stressful situations and may, therefore, be used as a coping strategy (5). In positive psychology, humor is viewed as a personal quality promoting resilience and well-being by means of cognitive reappraisal of stressful events (6). Humor has a broad range of effects on perceptions, attitudes, judgments, and emotions. In this regard, studies have associated humor and laughter with several physiological, psychological, sociological, and behavioral benefits for physical and mental health (2, 7–9).

## Functions of Humor

Based on the positive effects on health promotion and disease prevention, the question has been raised whether humor and laughter can be used in therapeutic settings (10). For example, humorous interactions between patients and service providers can support the therapeutic relationship (11, 12), and positively influence patients' experiences (11, 13). While the relationship between humor and health has been investigated through different perspectives, some areas are still under-researched (13). Communication studies, for instance, have primarily focused on the implications of mass media. In this context, it was observed that humor in advertisements enhances attention and improved the persuasiveness of preventive messages (14, 15). Whilst preventive health campaigns appeal to negative emotions like fear in order to dissuade their audience from hazardous health behavior, the use of fear can lead to defensive reactions, such as avoidance or denial. In order to circumvent the phenomenon whereby preventive messages are perceived as a threat to an individual's attitudinal freedom, introducing humor in health warnings may open the audience for arguments in favor of health. Because counterargument appears to be one of the most effective strategies used to resist persuasion (16), it remains an open question which effect the use of humor in health messages may have (15). However, humorous messages generally have the potential to reduce health-based anxiety and, in turn, promote positive behavior (17).

## Papers of the Research Topic

The Research Topic “*Cross-Disciplinary Perspectives on the Relationship Between Humor and Health: Theoretical Foundations, Empirical Evidence and Implications*” includes perspectives on the relationship between humor and health from various scientific disciplines, covering a variety of topics and samples (Table 1). From overall seven manuscripts published in this Research Topic (Markova et al.; Fischer et al.; Brigaud et al.; Baumeister and Fischer; Greve et al.; Bartzik et al.; Froehlich et al.), six used humor in an intervention study (Markova et al.; Fischer et al.; Brigaud et al.; Baumeister and Fischer; Bartzik et al.; Froehlich et al.), whereas one investigated humor as a moderating variable (Greve et al.). Half of the intervention studies used humor in the context of health communication (Fischer et al.; Brigaud et al.; Baumeister and Fischer; Froehlich et al.).

**Table 1:** Study characteristics.

	**Topic of interest**	**Humor**	**Sample**	**Sample size**
Markova et al.	Surgery	Clown intervention	Children (5–12 years)	62
Fischer et al.	Childhood vaccination	Fairytale	Parents of children at kindergarten	120
Brigaud et al.	Alcohol and tobacco prevention	Print health ads	Female undergraduate students	60
Baumeister and Fischer	Willingness to donate organs	Video sequences	University students	219
Greve et al.	Coping with unrequited love	Humor as moderating variable (sense of humor and humorous change of perspective)	General population	148
Bartzik et al.	Stress, flow experience, work enjoyment, and meaningfulness of work	Humor training	Nurses in training	104
Froehlich et al.	Preferences for (un)healthy food items	Video clips	General population	95

Among all studies, a variety of topics were covered: Some of them were directly related to healthcare provision, focusing either on a clown intervention for children undergoing surgery (Markova et al.) or on a humor intervention for nurses in training to reduce stress, and increase flow experience, work enjoyment, and perceived meaningfulness of work (Bartzik et al.); others focused on health-related factors such as nutrition (Froehlich et al.), alcohol and tobacco prevention (Brigaud et al.), organ donation (Baumeister and Fischer), childhood vaccination (Fischer et al.), and one further manuscript investigated humorous coping with unrequited love (Greve et al.). The study samples consisted of children undergoing surgery (Markova et al.), female undergraduate students (Brigaud et al.), university students (Baumeister and Fischer), parents of children at kindergarten (Fischer et al.), nurses in training (Bartzik et al.), and the general population (Greve et al.; Froehlich et al.).

## Future Research Directions

The short description of study characteristics shows the broad range of existing research related to humor. The well-conducted papers published in this Research Topic provide urgently needed evidence in this area and may, therefore, serve as a starting point for intensified research activities in the future. Until now, studies on humor and its implications on health are still a relatively new area of research. Thus, the current state of the art calls for greater research efforts employing careful theoretical formulations and sophisticated and rigorous methodological approaches. Despite widespread popular beliefs in the health benefits of humor and laughter, the research evidence for these effects is still quite weak, inconsistent and inconclusive (3, 18).

The major challenge for future research will be the operationalization of the complex and multidimensional construct of humor in the context of health-related activities. A clear distinction between humor, laughter and other (pleasant) emotions—and their link to health-related issues—is partially missing. Different components of humor are currently reflected in various conceptualizations. However, previous research has focused mainly on humor as a vague and oversimplified term (19), irrespective of further specifications, such as the style of humor. This may lead to biased and indistinct results when considering the effects of humor.

Further emphasis needs to be placed on the hypothesized mechanisms by which specific types of humor may affect health. It is essential to elaborate whether positive effects relate to physiological changes in the body caused by humor and laughter, effects from positive emotions (such as cheerfulness and optimism) (20), indirect effects from the alleviation of stressors or the proliferation of social support through the use of humor in interpersonal relationships (3).

We do hope that the studies published in this Research Topic provide new insights and underline the need for well-conducted studies dealing with the relationship between humor and health, going even beyond the topics, methods, and disciplinary perspectives covered in our call.

## Author Contributions

FF drafted the manuscript. CP and TS revised it critically for important intellectual content. All authors read and approved the final manuscript.

## Conflict of Interest

The authors declare that the research was conducted in the absence of any commercial or financial relationships that could be construed as a potential conflict of interest.

## Publisher's Note

All claims expressed in this article are solely those of the authors and do not necessarily represent those of their affiliated organizations, or those of the publisher, the editors and the reviewers. Any product that may be evaluated in this article, or claim that may be made by its manufacturer, is not guaranteed or endorsed by the publisher.

## References

[1] Fiacconi CM Owen AM. Using psychophysiological measures to examine the temporal profile of verbal humor elicitation. PLoS ONE. (2015) 10:e0135902. 10.1371/journal.pone.0135902 26332843 PMC4558035

[2] Martin RA. Humor, laughter, and physical health: methodological issues and research findings. Psychol Bull. (2001) 127:504–19. 10.1037/0033-2909.127.4.504 11439709

[3] Martin RA. Sense of humor and physical health: theoretical issues, recent findings, and future directions. Humor. (2004) 17:1–19. 10.1515/humr.2004.005

[4] Hehl F-J Ruch W. The location of sense of humor within comprehensive personality spaces: an exploratory study. Pers Indiv Differ. (1985) 6:703–15. 10.1016/0191-8869(85)90081-9

[5] Chapman AJ Foot HC. Humor and Laughter: Theory, Research, and Applications. Piscataway, NJ: Transaction Publishers (1996).

[6] Kuiper NA. Humor and resiliency: towards a process model of coping and growth. Eur J Psychol. (2012) 8:475–91. 10.5964/ejop.v8i3.464

[7] Bennett MP Lengacher C. Humor and laughter may influence health IV. Humor and immune function. Evid Based Complement Alternat Med. (2009) 6:159–64. 10.1093/ecam/nem149 18955287 PMC2686627

[8] Claxton-Oldfield S Bhatt A. Is there a place for humor in hospice palliative care? Volunteers say “Yes”! *Am J Hosp Palliat Care*. (2017) 34:417–22. 10.1177/1049909116632214 26917788

[9] Fry WF. Humor, physiology, and the aging process. In: Nahemow L McCluskey-Fawcett KA McGhee PE editors, Humor and Aging. Burlington: Elsevier (2013). p. 81–98. 10.1016/B978-0-12-513790-4.50011-X

[10] Moffat R. Is laughter a form of therapy? Med Leg J. (2013) 81:49. 10.1177/0025817213476396 23492896

[11] Haydon G van der Reit P Browne G. A narrative inquiry: humour and gender differences in the therapeutic relationship between nurses and their patients. Contemp Nurse. (2015) 50:214–26. 10.1080/10376178.2015.1021436 26073613

[12] Haydon G van der Riet P. A narrative inquiry: how do nurses respond to patients' use of humour? Contemp Nurse. (2014) 46:197–205. 10.5172/conu.2014.46.2.197 24787253

[13] McCreaddie M Payne S. Humour in health-care interactions: a risk worth taking. Health Expect. (2014) 17:332–44. 10.1111/j.1369-7625.2011.00758.x 22212380 PMC5060733

[14] Ahmed OH Lee H Schneiders AG McCrory P Sullivan SJ. Concussion and comedy: no laughing matter? PM R. (2014) 6:1071–2. 10.1016/j.pmrj.2014.08.945 25151359

[15] Blanc N Brigaud E. Humor in print health advertisements: enhanced attention, privileged recognition, and persuasiveness of preventive messages. Health Commun. (2014) 29:669–77. 10.1080/10410236.2013.769832 24160572

[16] Jacks JZ Cameron KA. Strategies for resisting persuasion. Basic Appl Soc Psych. (2003) 25:145–61. 10.1207/S15324834BASP2502_5

[17] Nabi RL. Laughing in the face of fear (of disease detection): using humor to promote cancer self-examination behavior. Health Commun. (2016) 31:873–83. 10.1080/10410236.2014.1000479 26652312

[18] Fritz HL Russek LN Dillon MM. Humor use moderates the relation of stressful life events with psychological distress. Pers Soc Psychol Bull. (2017) 43:845–59. 10.1177/0146167217699583 28903671

[19] Martin RA Ford T. The Psychology of Humor: An Integrative Approach. 2nd ed. San Diego, CA: Elsevier Science & Technology (2018).

[20] Pressman SD Cohen S. Does positive affect influence health? Psychol Bull. (2005) 131:925. 10.1037/0033-2909.131.6.925 16351329

